# Trends in accuracy management of continuous glucose monitoring systems

**DOI:** 10.1007/s13340-026-00901-w

**Published:** 2026-04-30

**Authors:** Keiichi Torimoto, Yosuke Okada

**Affiliations:** 1 First Department of Internal Medicine, School of Medicine, University of Occupational and Environmental Health, Kitakyushu, Japan; 2 Clinical Research Center, Hospital of the University of Occupational and Environmental Health, Kitakyushu, Japan

**Keywords:** Continuous glucose monitoring, Accuracy, Reliability, Error-grid analysis

## Abstract

Continuous glucose monitoring (CGM) has markedly advanced diabetes care by enabling real-time visualization of glycaemic variability, prevention of hypoglycaemia, and direct integration into therapeutic decision-making. As CGM use expands in routine practice and automated insulin delivery systems, however, accuracy has become a critical determinant of treatment safety. This mini-review summarizes recent advances in CGM accuracy management across three major driving forces: (1) accelerating technological innovation, including multi-analyte sensors, non-invasive devices, and artificial intelligence (AI)-based signal processing; (2) systematization and international harmonization of regulatory accuracy frameworks, exemplified by U.S. Food and Drug Administration (FDA) integrated CGM (iCGM) and the proposed CGM in Europe (eCGM) concept; and (3) growing societal demands for transparency, including public disclosure of performance data and strengthened lot-to-lot evaluation. We outline the four key dimensions of CGM accuracy—analytical accuracy, clinical accuracy, trend accuracy, and precision. We then review the evolution of regional accuracy standards, focusing on highly influential frameworks in the United States and Europe. Key considerations in accuracy-study design are discussed, along with clinical risks associated with reduced accuracy optimization, and progress toward global standardization. Finally, we examine future directions in the era of next-generation technologies, such as multi-analyte and non-invasive sensors, AI-driven accuracy optimization, and progress toward international standardization. This review provides an overview of the current landscape and future directions of CGM accuracy management in an era where fluctuations in accuracy directly affect treatment safety. We aim to clarify the perspectives required in both clinical practice and research to ensure safe and effective use of CGM.

## Introduction

The widespread adoption of continuous glucose monitoring (CGM) has fundamentally transformed clinical practice by enabling visualization of glucose trends, quantification of glycaemic patterns, optimization of hypoglycaemia prevention, and individualized treatment adjustments. Today, CGM-derived values are no longer mere “measurements”; they represent core data directly used for automated insulin delivery (AID) systems, insulin dose adjustments, and clinical risk assessment. Consequently, CGM accuracy has shifted from being a technical characteristic to a foundation that effectively defines “treatment safety.”

With the rapid evolution of CGM technology, challenges related to accuracy have become increasingly apparent. In the current era of AID, even a seemingly small error of 15–20 mg/dL may lead to clinically meaningful consequences, such as unnecessary carbohydrate intake, excessive insulin dosing, or missed hypoglycaemia. In addition, the global expansion of the CGM market has promoted the entry of lower-cost devices, while regulatory frameworks still differ substantially across regions. This heterogeneity further amplifies variability in device accuracy and reliability.

**Fig. 1:**
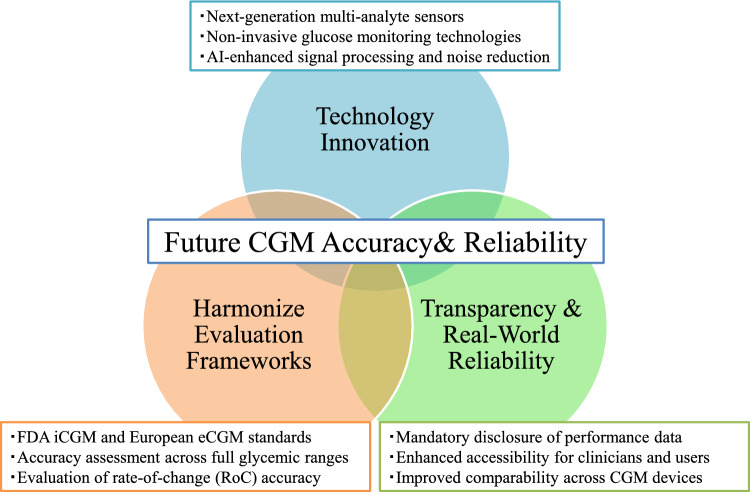
Three major driving forces shaping the evolution of CGM accuracy management. CGM accuracy is influenced by three interacting forces: advances in core sensing and AI-based signal-processing technologies; systematization and international harmonization of accuracy-evaluation frameworks; and enhanced data transparency that enables objective comparison of device performance. Together, these elements define the emerging landscape of high-reliability CGM systems

To understand this “variability in accuracy,” it is useful to consider three major driving forces that shape CGM accuracy (Fig. 1). These include: (1) accelerating technological innovation, such as multi-analyte sensors, non-invasive devices, and artificial intelligence (AI)-based signal correction; (2) systematization and international standardization of accuracy evaluation frameworks, symbolized by U.S. Food and Drug Administration (FDA) iCGM, the proposed CGM in Europe (eCGM) concept, and ongoing initiatives by International Federation of Clinical Chemistry and Laboratory Medicine (IFCC) and International Organization for Standardization (ISO); and (3) increasing demands for transparency of device performance and real-world data, which promote objective product comparison. The interaction among these three forces defines the emerging landscape of next-generation CGM accuracy management.

Against this background, appropriate management and evaluation of CGM accuracy have become more important than ever. In this review, we summarize the multidimensional components of CGM accuracy, the evolution of international accuracy standards, methodological issues in accuracy evaluation studies, the clinical impact of poor accuracy, and future directions for accuracy management.

## CGM accuracy: four pillars

CGM accuracy is inherently multidimensional and cannot be fully described by a single numerical metric. Unlike self-monitoring of blood glucose (SMBG), which provides discrete capillary glucose measurements, CGM continuously measures glucose in interstitial fluid and estimates blood glucose via device-specific algorithms. This process introduces multiple potential sources of error. CGM accuracy can be more easily understood by considering four axes [1]:

### Analytical accuracy

Analytical accuracy describes the degree of agreement between CGM values and reference blood glucose measurements. It is typically assessed using indices such as absolute relative difference (ARD), mean absolute relative difference (MARD), and the proportion of values within ± 15 mg/dL or ± 15% of reference, among others. Analytical accuracy varies according to glucose range, sensor insertion site, sensor wear duration, and inter-individual differences.

### Clinical accuracy

**Fig. 2:**
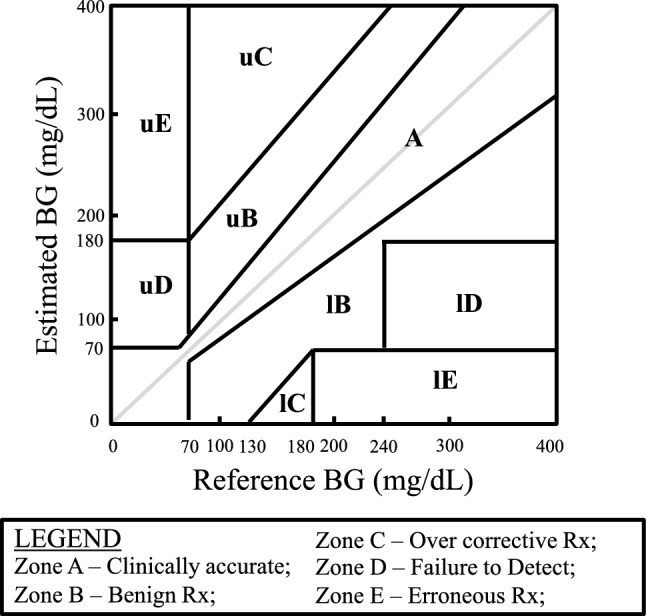
Overview of clinical accuracy evaluation using Error Grid Analysis. This figure summarizes the principal error grid frameworks used to assess the clinical accuracy of glucose-measuring systems. The Clarke Error Grid classifies paired CGM–reference glucose values into zones that correspond to different levels of clinical risk arising from potential treatment decisions. In the figure, “u” and “l” denote the upper and lower boundaries of each zone, respectively (e.g., uB = upper boundary of Zone B; lB = lower boundary of Zone B)

Clinical accuracy evaluates how CGM errors affect treatment decisions. The most widely used approach is error grid analysis (Clarke [2], Consensus [3], Surveillance [4], and continuous glucose error-grid analysis [CG-EGA] [5]), which classifies paired values into zones according to their potential clinical risk. Error grids delineate zones ranging from a “safe” zone (Zone A), where deviations do not affect treatment decisions, to “dangerous” zones (Zones C–E), where errors may lead to inappropriate treatment (Fig. 2). Even when analytical accuracy appears good, clinically dangerous errors may still occur; thus, distinguishing between analytical and clinical accuracy is essential.

For example, when the reference glucose value is 310 mg/dL and the CGM reading is 400 mg/dL, the relative error is+30%, but this pair remains in the safe Zone A in the Clarke error grid. In contrast, when the reference is 65 mg/dL and the CGM reading is 80 mg/dL, the relative error is− 30%, which falls into Zone D, indicating potentially dangerous misclassification (Fig. 2). Understanding this difference between analytical and clinical accuracy is crucial in interpreting CGM performance.

### Trend accuracy

Trend accuracy [6] represents the ability of CGM to correctly capture the direction (rising or falling) and rate of change (RoC) of glucose levels. It is particularly important in dynamic situations such as postprandial hyperglycaemia, exercise-induced glucose decline, and recovery from hypoglycaemia, where rapid changes in glucose pose significant clinical risks.

### Precision

Precision reflects the reproducibility of CGM readings, typically assessed by simultaneously wearing multiple sensors in the same individual. It provides information on lot-to-lot variability and performance changes over the sensor wear period.

These four components are complementary and jointly determine the overall clinical value of a CGM system.

## Regulatory harmonization

With the global spread of CGM, regulatory authorities in each region have begun to establish and refine accuracy standards. This section focuses on the highly influential frameworks in the United States and Europe.

### United States: FDA iCGM criteria

In 2018, the US Food and Drug Administration (FDA) introduced a new regulatory category, “integrated CGM (iCGM)” [7]. Only CGM systems that meet stringent criteria for accuracy, reliability, and connectivity are authorized as iCGM. iCGM devices are approved for non-adjunctive use, allowing CGM values to be used directly for treatment decisions without confirmatory fingerstick measurements.

Key elements of the iCGM special controls for accuracy include:

1. Accuracy criteria
  Time below range (TBR; < 70 mg/dL): ≥ 85% of values within ± 15 mg/dL
  Time in range (TIR; 70–180 mg/dL): ≥ 70% of values within ± 15%
  Time above range (TAR; > 180 mg/dL): ≥ 80% of values within ± 15%
  Very high glucose range: ≥ 99% of values within ± 40%
  These criteria primarily correspond to analytical accuracy and clinical accuracy.
2. Rate-of-change performance
  Misclassification rate for glucose rate-of-change < 1% (to limit false detection of rapid increases or decreases).
    This reflects trend accuracy.
3. Durability of performance
  Maintenance of accuracy over the entire approved sensor wear period.
    This corresponds to precision.
4. Robustness across conditions
  Evaluation across multiple manufacturing lots and multiple sensor insertion sites.
    This also reflects precision.
5. Connectivity and data integrity
  Real-time connectivity and appropriate handling of data gaps.
6. Paediatric data
  Submission of paediatric performance data or a justified rationale for exemption.
    These requirements currently represent the most stringent CGM accuracy criteria worldwide and are effectively regarded as a de facto global standard.

### Europe: Conformité Européenne (CE) marking and the eCGM proposal

CE marking is required for marketing medical devices in Europe; however, historically, no CGM-specific accuracy standards were defined within this framework. Consequently, there was limited obligation to disclose detailed performance data, and reference methods or study designs were not standardized. In some cases, such as reports from the Campania Region in Italy, CGM systems bearing a CE mark were found to exhibit suboptimal accuracy in real-world use.

In response to these concerns, European experts have proposed a new minimum performance standard referred to as “eCGM” [1]. This concept draws heavily on the FDA iCGM framework and represents an important step toward strengthening CGM accuracy management across Europe.

### International harmonization: IFCC and ISO initiatives

The International Federation of Clinical Chemistry and Laboratory Medicine (IFCC) and the International Organization for Standardization (ISO) are working toward international standardization of CGM accuracy evaluation, including the development of CGM-specific measurement standards and test protocols [8]. As international harmonization progresses, regional discrepancies in CGM accuracy criteria are expected to diminish, increasing the likelihood that patients worldwide will have access to data of comparable quality.

## Importance of study design in accuracy evaluation

The reliability of CGM accuracy data depends heavily on study design [9]. Selection of the study population is particularly important. Studies that include only patients with relatively stable glucose levels may overestimate accuracy. Inclusion of populations with greater glycaemic variability, such as individuals with type 1 diabetes and children, provides a more realistic assessment of clinical performance.

Adequate paired reference data across the full glycaemic spectrum—including hypoglycaemia (< 70 mg/dL) and marked hyperglycaemia (> 300 mg/dL)—are essential. Under dynamic conditions, especially during meals, insulin corrections, and physical activity, errors are more likely to occur, and rate-of-change accuracy becomes critically important. Laboratory-based reference measurements (e.g., YSI analysers) are recommended; studies that use SMBG as the reference may yield biased accuracy estimates due to inherent SMBG error.

In addition, evaluation should cover the entire approved sensor wear period, include multiple insertion sites, and assess at least three manufacturing lots to capture lot-to-lot variability.

Transparency of accuracy data is indispensable. Proprietary, non-public company data are insufficient for clinicians to judge real-world performance. Instead, accuracy assessments based on peer-reviewed, publicly available studies provide the most reliable foundation for scientific and clinical discussion of CGM performance.

## Clinical consequences of poor accuracy

Impaired CGM accuracy can have direct and serious consequences for treatment decision-making. For example, if CGM readings are falsely low while actual glucose levels are within the normal range, patients may fear hypoglycaemia and ingest unnecessary carbohydrates. Conversely, falsely elevated readings (“pseudo-hyperglycaemia”) may prompt excessive insulin dosing, particularly hazardous in insulin-treated patients, where it may directly lead to hypoglycaemia.

Misinterpretation of glucose dynamics can be equally harmful. If CGM fails to adequately capture a downward trend, impending hypoglycaemia may be missed and preventive actions delayed. Conversely, overestimation of rising glucose may trigger unnecessary correction insulin. Errors in trend information can therefore have even more serious clinical implications than static point-in-time errors.

In this way, poor CGM accuracy is not simply a measurement issue; it can drive inappropriate therapeutic interventions, increase glycaemic variability, and raise the risk of clinically significant hypoglycaemic and hyperglycaemic events. Selecting CGM systems with high accuracy is thus essential to maintain both the quality and safety of diabetes management.

International guidelines [10] clearly recommend confirmatory capillary blood glucose measurements when CGM readings do not match symptoms. It is well established that certain conditions—including rapidly changing glucose levels, the first day of sensor wear, and substantial mechanical or physical interference—are associated with increased CGM error. Therefore, even for iCGM systems, clinical practice should still include fingerstick confirmation in specific high-risk situations.

## Future directions

CGM technology has advanced rapidly in recent years, and accuracy management is entering a new phase. One notable development is the emergence of next-generation multi-analyte sensors [11] that measure multiple biomarkers simultaneously. Real-time monitoring of metabolic markers such as lactate and ketone bodies, in addition to glucose, may enable earlier detection of metabolic dysregulation and further improvements in treatment quality. However, multi-analyte sensors raise new concerns regarding cross-interference and signal noise, necessitating even more stringent accuracy management.

Development of non-invasive CGM [12] is also progressing. Devices using optical or spectroscopic techniques are attractive because they avoid the pain associated with skin puncture. Currently, however, their performance remains sensitive to environmental factors and skin condition, and issues regarding accuracy, stability, and time lag remain unresolved. Widespread clinical use of non-invasive CGM will require construction of an accuracy management framework based on principles that differ from those applied to invasive sensors.

At the same time, artificial intelligence (AI)-based signal processing [13] is opening new possibilities for improving CGM accuracy. Techniques such as machine-learning–based noise reduction, outlier correction, and real-time algorithmic adjustment under dynamic conditions are moving into practical use. These approaches extend the concept of accuracy from purely sensor-intrinsic performance to “algorithm-enabled optimization of accuracy.”

Efforts to unify international accuracy standards are also accelerating. In addition to FDA iCGM and the proposed European eCGM concept, IFCC and ISO are pursuing standardization initiatives, increasing the likelihood that CGM accuracy evaluation methods will eventually be harmonized worldwide. Such standardization would help ensure a minimum level of sensor performance for patients regardless of geographic region, with significant implications for global health.

Finally, transparency of sensor accuracy data is expected to become increasingly important. Mandatory disclosure of performance data would facilitate objective comparison of products by healthcare professionals and users, while also providing strong incentives for manufacturers to improve quality. Enhanced transparency is likely to contribute to healthy market growth and greater trust in CGM technologies.

Overall, CGM accuracy management is evolving at the intersection of three major trends: technological advancement of sensors, systematization of evaluation methods, and improvement of data transparency. The interplay of these forces is expected to shape the future of CGM (Fig. 1).

## Conclusion

CGM accuracy management is a critical foundation for ensuring the safety and quality of diabetes care. Stringent criteria, typified by FDA iCGM requirements, are increasingly becoming international benchmarks, while European initiatives such as the eCGM proposal signal a growing commitment to accuracy improvement. Advances in accuracy evaluation methodology, data transparency, next-generation sensors, and AI-driven signal processing are expected to further enhance the clinical value of CGM. As CGM continues to evolve, ongoing efforts toward standardization and high-accuracy performance will be essential to support safer and more reliable use of CGM in daily clinical practice.
